# Autogenous Dentin Graft in Bone Defects after Lower Third Molar Extraction: A Split-Mouth Clinical Trial

**DOI:** 10.3390/ma13143090

**Published:** 2020-07-10

**Authors:** Luis Sánchez-Labrador, María Martín-Ares, Ricardo Ortega-Aranegui, Juan López-Quiles, José María Martínez-González

**Affiliations:** Department of Dental Clinical Specialities, Faculty of Dentistry, Complutense University of Madrid (UCM), Plaza Ramon y Cajal S/N, 28040 Madrid, Spain; luissanc@ucm.es (L.S.-L.); mmartinares@gmail.com (M.M.-A.); riortega@ucm.es (R.O.-A.); jlopezquiles@odon.ucm.es (J.L.-Q.)

**Keywords:** autogenous dentin, dentin graft, surgical extraction, third molar surgery

## Abstract

Various biomaterials are currently used for bone regeneration, with autogenous bone being considered the gold standard material because of its osteogenic, osteoconductive, and osteoinductive properties. In recent years, the use of autogenous dentin as a graft material has been described. This split-mouth clinical trial assesses the efficacy of autogenous dentin for the regeneration of periodontal defects caused by bone loss associated with impacted lower third molar extraction. Fifteen patients underwent bilateral extraction surgery (30 third molars) using dentin as a graft material on the test side, and leaving the control side to heal spontaneously, comparing the evolution of the defects by evaluating probing depth at three and six months post-operatively. Bone density and alveolar bone crest maintenance were also evaluated six months after surgery, and pain, inflammation, mouth opening capacity on the second and seventh days after surgery. Probing depth, radiographic bone density, and alveolar bone crest maintenance showed significant differences between the test and control sides. Autogenous dentin was found to be an effective biomaterial for bone regeneration after impacted lower third molar extraction.

## 1. Introduction

Impacted lower third molar (LTM) extraction surgery, whether prophylactic or therapeutic, is a very common procedure in the field of oral and maxillofacial surgery [1]. The frequency of impaction lies between 66% and 77% [2]. In addition to its high prevalence, third molar extraction is a surgical procedure accompanied by complications that include periodontal problems characterized by bone loss on the distal aspect of the second molar [3,4].

Numerous authors [5,6,7] support the use of regenerative techniques as complimentary treatments, showing that the use of bone substitutes with resorbable or non-resorbable membranes is an effective method for healing post-extraction defects in comparison with extraction without biomaterial grafting.

Different biomaterials have been used to stimulate or improve bone gain at these post-extraction sites. Nevertheless, autogenous bone continues to be considered the material of choice for bone regeneration, as it is the only option that fulfills the criteria of osteogenesis, osteoconduction, and osteoinduction [8,9]. Nevertheless, it suffers several disadvantages due to its limited availability and associated morbidity at the donor site [10].

In recent years, dentin has been investigated as a possible biomaterial for bone regeneration. In 2010, Kim et al. [11] described the first ever use of dentin in implant dentistry, obtaining the dentin from the patient’s extracted teeth. Autogenous dentin possesses ideal physical (density, roughness, and homogeneity) and chemical (composed of calcium/phosphate similar to human bone in cortical areas) properties and was found to behave effectively as a biocompatible material that stimulated bone tissue formation.

Human dentin is composed of 70% organic content with four types of calcium phosphate (hydroxyapatite, tricalcium phosphate, octacalcium phosphate, and amorphous calcium phosphate), which provide dentin with its osteoconductive properties. The hydroxyapatite in dentin is in the form of calcium phosphate with low crystal content, which makes it easily degradable by osteoclast activity [11]. It is composed of 20% organic content, of which 90% is a type I collagen network and 10% non-collagenous proteins (osteocalcin, osteonectin, sialoprotein, and phosphoprotein, which participate in bone calcification) and growth factors (bone morphogenetic proteins: BMPs, and insulin-like growth factor, which give the tooth osteoinductive properties); the remaining 10% is water [12,13]. Nevertheless, it does not possess the osteogenic capacity of autogenous bone, and its quantity is also limited, depending on the condition of discarded teeth [14].

Following the study published by Kim et al. [11], other researchers have used dentin for bone regeneration in implant dentistry in the context of alveolar preservation in sinus lifting [15,16,17,18,19].

In 2015, a unique clinical case report described the use of autogenous dentin for alveolar preservation of an upper right third molar socket, observing bone formation in the grafted area in periapical radiographs and micro-computerized tomography over a one-year follow-up (micro-CT) [20].

A systematic review by Gual-Vaqués et al. [14] highlighted the positive results obtained with this graft material, used in the form of granules for sinus lifting and bone regeneration, observing new bone formation with the presence of osteoblasts and osteoclasts around the graft material. The authors also expressed a need for further long-term studies with larger sample sizes. Another systematic review by Ramanauskaite et al. [21] investigated the use of dentin for sinus lifting and bone regeneration, again highlighting the need for further clinical trials of dentin grafting.

Therefore, the aim of this clinical trial was to use autogenous dentin to reduce the probing depth (PD) and improve clinical insertion levels in cases of impacted third molar extractions, comparing the clinical and radiographic results with the conventional surgical approach.

## 2. Materials and Methods

### 2.1. Study Design

This randomized, split-mouth clinical trial was conducted at the Oral Surgery Service of the Faculty of Dentistry at the Complutense University of Madrid (UCM). The study followed Declaration of Helsinki principals for research involving human subjects, and all patients provided their informed consent to take part. The study protocol was approved on 25 May 2018, by the Scientific Committee for Clinical Trials at the San Carlos Hospital, Madrid (Spain) (Reg. N° 18/203-E); patients were recruited over a period of 12 months after approval was granted.

### 2.2. Participants

A total of 15 patients were selected who were attending the Oral Surgery Service at the UCM Faculty of Dentistry presenting indications for bilateral lower third molar extraction. The main researcher (L.S.L.) assessed the patients, who were provided with full information about the purpose of the trial, the procedures involved, and the possible risks and benefits associated with the treatment. Patients then signed an informed consent form before the start of the trial. A medical and dental history was created for each patient including a panoramic radiograph. In cases presenting a close relationship between LTM and inferior alveolar nerve (IAN), a cone beam computerized tomography scan (CBCT) was taken. 

Inclusion and exclusion criteria were as follows: 

#### 2.2.1. Inclusion Criteria

- Patients of either sex aged between 18 and 25 years.
- Indications for bilateral extraction of impacted LTMs.
- Presence of lower second molars.
- Patients in good health (ASA I or II).
- Patients able to understand and carry out instructions given by the researchers.

#### 2.2.2. Exclusion Criteria

- Pregnant or lactating women.
- Patients in treatment with nonsteroidal anti-inflammatory drugs (NSAIDs).
- Patients with periodontal diseases.

### 2.3. Sample Size, Blinding, and Randomization

The trial included 15 patients (N = 15), who were intervened bilaterally (30 impacted LTMs). On the test side after LTM extraction, dentin from the extracted LTM was used as a graft material. On the control side, the alveolus was left to heal in the conventional way by forming blood coagulate.

In order to determine sample size, a pilot study was performed, based on the primary outcome parameter (probing depth). This study was carried out at the Oral Surgery Service of Complutense University of Madrid, enrolling 5 patients (10 alveoli), with a split-mouth design, obtaining a mean reduction on probing depth of 2.4 ± 1.09 mm and 1 ± 0.66 mm, in test and control side, respectively. Using G Power 3.1 (Dusseldorf, Germany), considering an alpha-type error of 5% and a beta-type error of 5%, the estimation resulted in 10 patients per group. Patients’ number was ampliated to 15, to prevent possible dropouts of patients during the follow-up.

A single clinician (L.S.-L.) performed all surgeries bilaterally. Randomization was performed by another researcher (J.M.M.-G.), using opaque envelopes whose content stipulated group assignation of the extraction sites (as test side or control side).

As the test group required longer surgical time than the control group (due to the extra time needed to prepare the graft material) the patients could not be blinded.

### 2.4. Surgical Procedure

All interventions were performed by the same surgeon (L.S.-L.). Local anesthetic was administered consisting of 4% articaine and 1:100,000 adrenaline for the inferior alveolar, lingual, and buccal nerves (Ultracaine^®^, Normon SL, Madrid, Spain).

An intrasulcular incision was made from the distal face of the first lower molar, passing the lower second molar, creating an oblique flap on the disto-vestibular surface in the direction of the mandibular ascending ramus. After mucoperiosteal release of the vestibular flap and slight release of the lingual flap taking care to protect the lingual nerve, the phases of LTM extraction began with osteotomy to free bone blocks, followed by coronal-radicular odontosection if necessary, and extraction of the tooth fragments, curettage of the distal surface of the second molar, and socket washing.

**Figure 1:**
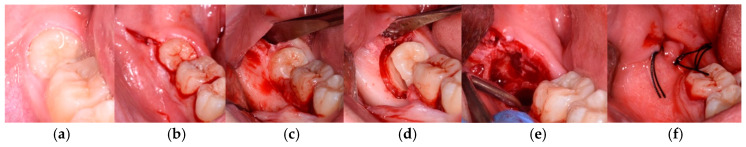
Treatment sequence on control side. (**a**) Baseline. (**b**) Incision. (**c**) Mucoperiosteal release. (**d**) Osteotomy and odontosection. (**e**) Revision. (**f**) Suture.

On the control side, after inspecting and washing the socket, the mucoperiosteal flap was repositioned, sutured with 4/0 silk (Aragó^®^, Barcelona, Spain) and compressed with gauze (Figure 1).

On the test side, after inspecting and washing the socket, the site was compressed with gauze soaked in 0.12% chlorhexidine (PerioAid^®^, Dentaid, Barcelona, Spain), while the clinician prepared the graft material. The extracted tooth, whether complete or split by odontosection, was cleaned with sterile gauze to remove traces of soft tissue and then dried with compressed air. Afterwards, the tooth was placed in a dentin grinding device (Smart Dentin Grinder^®^, KometaBio, BIONER, Barcelona, Spain) and ground for 3 s following the manufacturer’s instructions. When ground, it was sieved for 20 s to obtain a particle size of 300–1200 µm. The material obtained was mixed with 0.5 molar sodium hydroxide and 20% ethanol for 12 min in a sterile flask to eliminate any organic remains, bacteria, or toxins present in the dentin. Lastly, the particles were submerged in saline for 3 min, removing excess saline with a sterile pipette.

**Figure 2:**
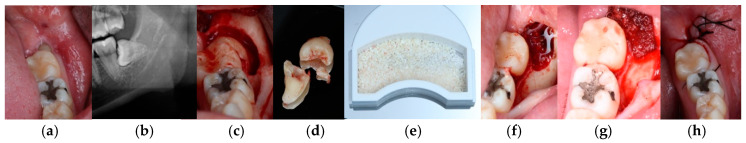
Treatment sequence on test side. (**a**) Baseline. (**b**) Panoramic radiograph. (**c**) Osteotomy. (**d**) Tooth fragments. (**e**) Graft material. (**f**) Graft material in the alveolus. (**g**) Fibrin sponge over the graft. (**h**) Suture.

The graft material was transported to the vacant alveolus using sterile plastic instruments without pressure, carefully placing the material on the distal surface of the lower second molar’s distal root. When this was done, a fibrin sponge (Gelatamp^®^, Coltene, Langenau, Germany) was placed over the graft before suturing the wound with 4/0 silk (Aragó^®^, Barcelona, Spain) (Figure 2).

After surgery, patients were prescribed an anti-inflammatory (25 mg Dexketoprofen every 8 h for 4 days) and a rescue analgesic (650 mg Paracetamol every 8 h) to be taken in case of pain. Antibiotics were just administered in cases of post-operative infection.

The test side and control side were treated separately with an interval of 4 weeks between the two interventions.

### 2.5. Evaluation of Outcomes

#### 2.5.1. Pre-Operative Variables

**Figure 3:**
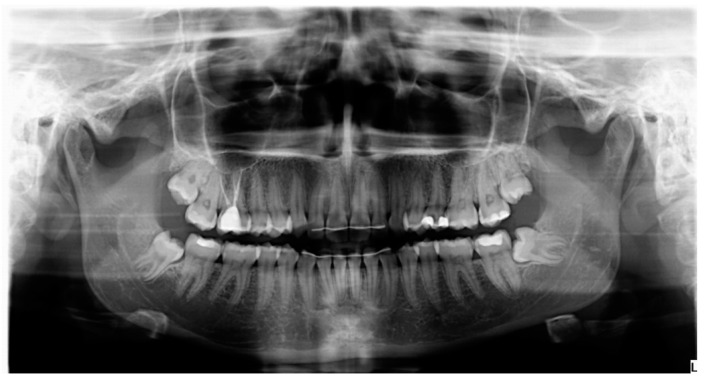
Pre-operative panoramic radiograph.

At the first visit, the main researcher (L.S.-L.) recorded the following variables: age; sex; medical and dental history; location and position of the LTMs; the close or otherwise relation with the inferior alveolar nerve observed in a panoramic radiograph (Figure 3).

**Figure 4:**
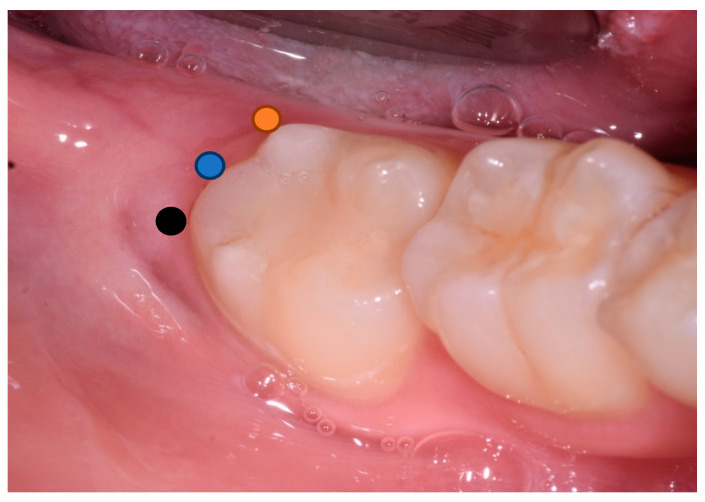
Pre-operative photograph showing disto-vestibular (DV) (black); disto-medial (DM) (blue); and disto-lingual (DL) (orange) points where probing depth (PD) was evaluated.

Pre-operative PD on the distal aspect of the lower second molar measured at three points: Disto-vestibular (DV), disto-medial (DM), and disto-lingual (DL) (Figure 4); gingival recession and insertion level before surgery. 

Radiographic exploration was performed with a panoramic radiograph, and just in cases showing a close relation between an LTM and the inferior alveolar nerve, a pre-operative CBCT was taken.

Facial patterns and mouth opening capacity were measured:

- Facial measurements were taken with silk thread measuring trago-pogonion distance, external labial commissure to the tragus, and from the gonion to the external canthus of the eye, following Amin and Laskins’ modified criteria for evaluating post-operative inflammation [22].
- Mouth opening capacity was measured with an analogue caliper (Acha^®^, Eibar, Spain), measuring a straight line from upper incisor edge to lower incisor edge to subsequently evaluate reductions in post-operative mouth open capacity.

#### 2.5.2. Intra-Operative Variables

The following intra-operative variables were recorded: insertion level, surgical time (measured from the start of incision to the last suture), and the surgical difficulty index according to the modified Parant scale [23].

**Figure 5:**
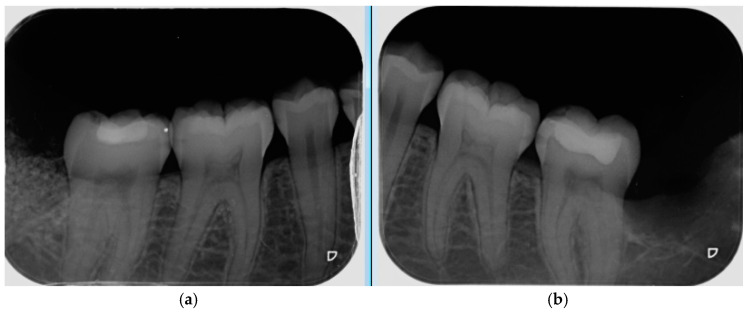
Intra-operative radiographs taken after autogenous dentin graft placement on study side (**a**) and Spontaneous healing with blood coagulate on control side (**b**).

A periapical radiograph was taken to check adequate graft material placement in the socket (Figure 5), in comparison with spontaneous healing on the control side.

#### 2.5.3. Post-Operative Variables

All patients were examined by the same researcher who had performed surgery (L.S.L.). Pain level, inflammation, mouth opening capacity, probing depth, and bone density were recorded to evaluate differences in relation to the use or non-use of the graft material.

**Figure 6:**
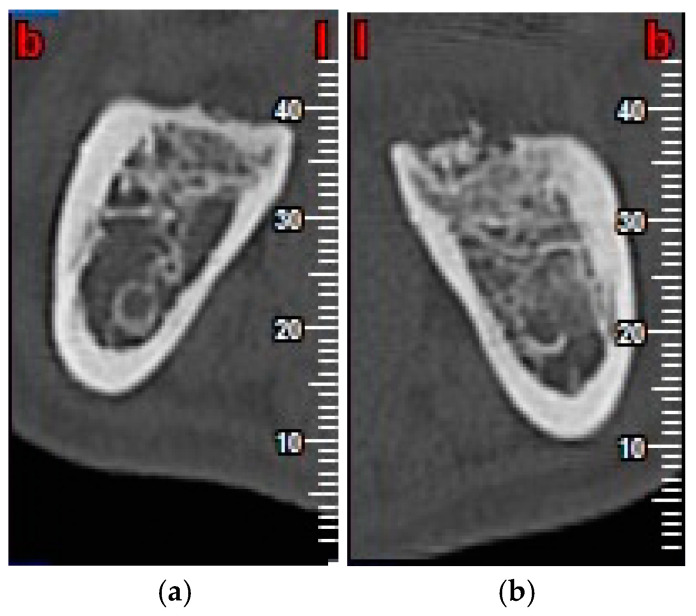
Cone beam computerized tomography scan (CBCT) scan, taken six months after lower third molar (LTM) extraction, in control group (**a**) and test group (**b**).

**Figure 7:**
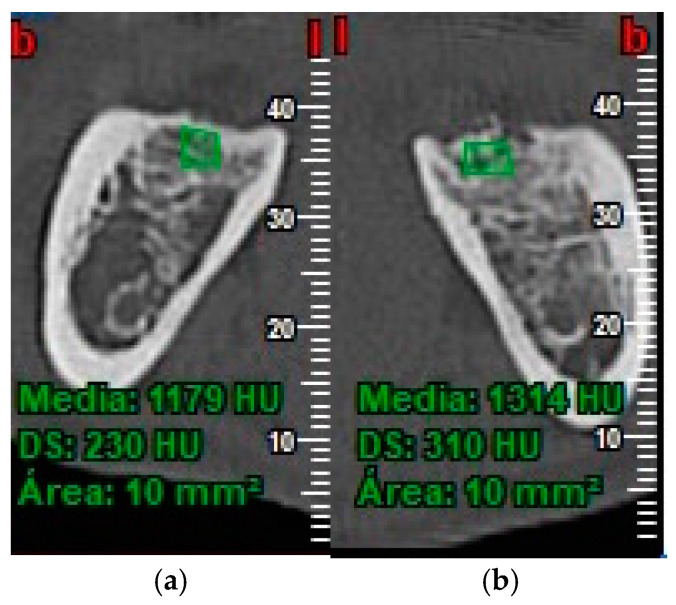
Bone density in control group (**a**) and test group (**b**).

**Figure 8:**
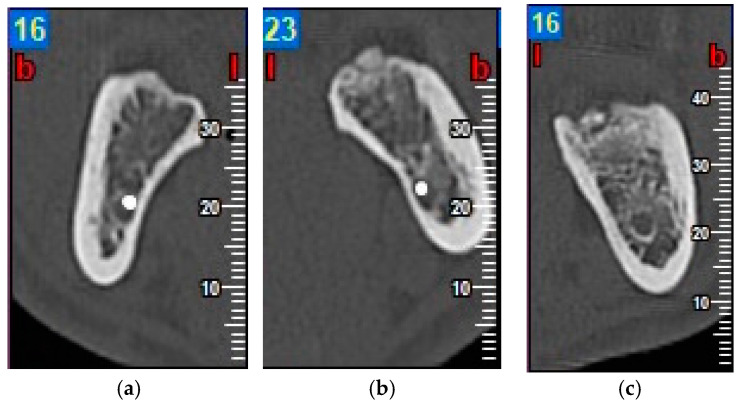
Degree of corticalization: complete (**a**); incomplete (**b**); and absent (**c**).

**Figure 9:**
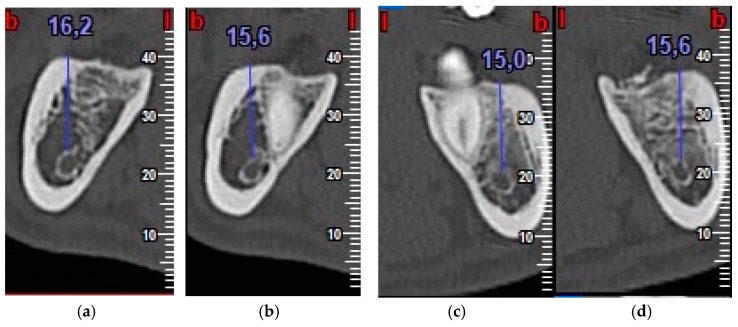
Distance from inferior alveolar nerve (IAN) to bone crest on control side (**a,b**) and test side (**c,d**).

- Inflammation was evaluated by measuring facial patterns 48 h and 1 week after surgery. Mouth opening capacity was measured at the same times with an analogue caliper.
- Pain was evaluated using a visual analogue scale (VAS) scored as 1–10, 1 representing minimal pain and 10 maximum pain, every day for 7 days after extraction surgery. The number of rescue analgesics taken (1, 2 or 3 per day) during the 7 days after surgery was recorded.
- Probing depth was measured 3 and 6 months after surgery at the DV, DM and DL points on the distal aspect of the second molar as shown in Figure 3.
- Bone density, the degree of corticalization, and crestal bone height maintenance were measured 6 months after surgery on CBCT sagittal slices in all patients (Figure 6), using the same equipment (Newtom VGI evo, QR srl-Verone, Italy).
- Bone density was measured in Hounsfield units (HU) (Figure 7).
- The degree of corticalization was classified as complete (cortical ≥ 1 mm), incomplete, or absent (Figure 8).
- Distance from the IAN to the bone crest was measured at the distal aspect of the lower second molar, at the center of the third molar alveolus (Figure 9).

### 2.6. Statistical Analysis

Data were entered on a spreadsheet (MS Excel 2007, Microsoft Inc., Redmond, WA, USA) at the end of the trial and analyzed with statistical software (SPSS, version 17.0, Chicago, IL, USA) by a statistician independent from the trial.

This split-mouth trial compared the use or non-use of autogenous dentin grafts, whose effects were evaluated by evaluating probing depth, radiographic bone density, alveolar crest maintenance, and degree of corticalization. Pain, inflammation and mouth opening capacity were also compared.

Descriptive statistics were calculated for all variables (frequency, mean, standard deviation). Qualitative variables were analyzed with the chi-squared test. Quantitative variables (probing depth, radiographic bone density, alveolar crest maintenance, facial perimeter, mouth opening, post-operative pain, rescue analgesics taken) were analyzed with Student’s T-test and the Mann-Whitney test.

For all results, a 95% confidence interval was registered (significance level *p* < 0.05).

## 3. Results

Fifteen patients underwent bilateral surgery between September 2018 and September 2019, making a total of 30 impacted LTM extractions. The patients had a mean age of 21.86 years and included four men (26.66%) and 11 (73.34%) women. The trial’s follow-up took place six months after LTM extraction; all patients completed the follow-up period.

All the LTMs were extracted successfully. Autogenous dentin was placed in 15 alveoli as a graft material, while the other 15 alveoli healed spontaneously (blood coagulate). Information about LTM position and situation, intraoperative difficulty, and surgical time are shown in Table 1 and Table 2.

### 3.1. Probing Depth (PD)

**Figure 10:**
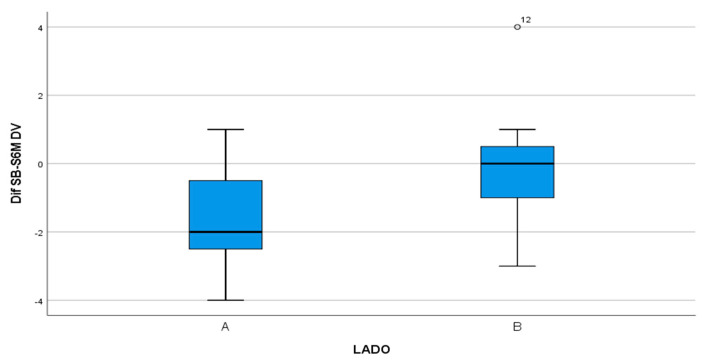
Box-plot representing the difference in PD between the test group (A) and control group (B) on the DV surface.

Comparing reductions in PD between the test and control groups from the start of the trial to the end of the six-month follow-up, test group DV surfaces obtained a mean reduction of 1.53 mm compared with 0.27 mm in the control group (Figure 10) with statistically significant difference (*p* = 0.038).

**Figure 11:**
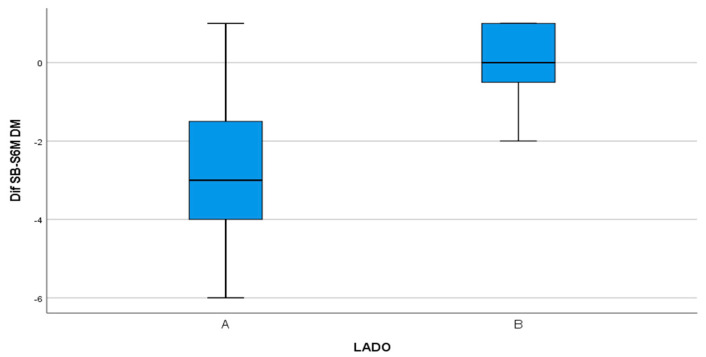
Box-plot representing the difference in PD between the test group (A) and control group (B) on the DM surface.

On the DM surface, the test group underwent a reduction of 2.8 mm, compared with 0.07 mm in the control group (Figure 11) with statistically significant difference (*p* < 0.001).

**Figure 12:**
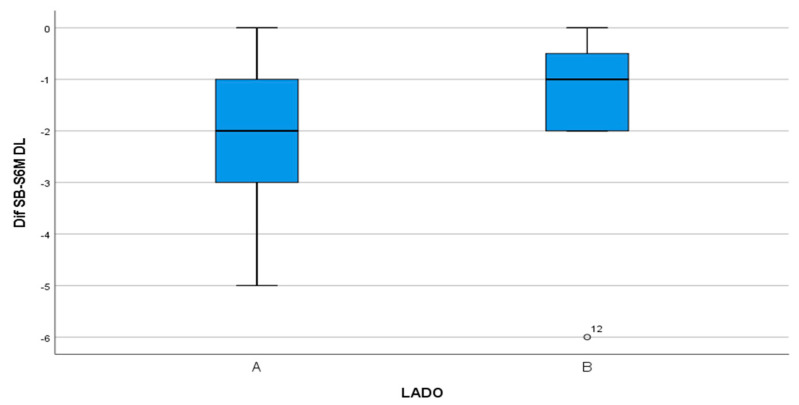
Box-plot representing the difference in PD between the test group (A) and control group (B) on the DL surface.

The DL surface obtained a reduction of 2.33 mm in the test group compared with 1.4 mm in the control group (Figure 12) but without statistically significant difference (*p* = 0.109).

**Figure 13:**
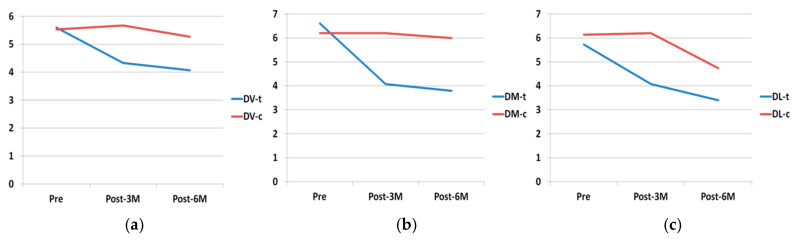
Evolution of PD on DV (**a**), DM (**b**), DL (**c**) surfaces. t: test; c: control.

In this way, the overall evolution of PD underwent a significant reduction in the test group compared with the control group. This reduction was mainly produced during the first three months after surgery, as shown in Figure 13.

### 3.2. Bone Density

**Figure 14:**
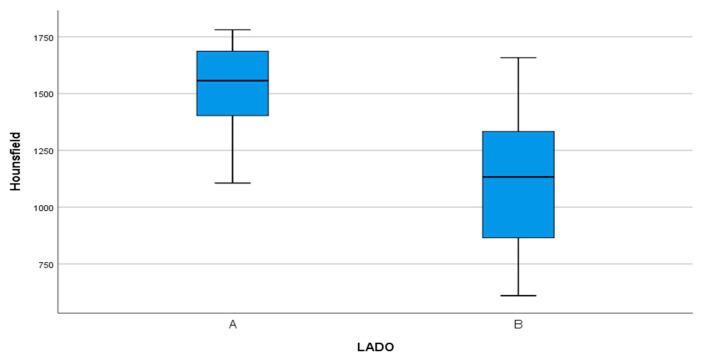
Bone density measured in Hounsfield units in test group (A) compared with control group (B).

Evaluated by CBCT scans taken six months after surgery, bone density (Hounsfield units: HU) was greater in the test group compared with the control group, as shown in Figure 14, with statistically significant difference (*p* < 0.001).

### 3.3. Degree of Corticalization

In the test group, 20% of patients presented an absence of corticalization compared with 0% in the control group; 60% presented incomplete corticalization in the test group compared with 40% in the control group; and 20% presented complete corticalization in the test group compared to 60% in the control group. However, the Student’s t-test did not find statistically significant differences between the groups.

### 3.4. IAN-Crestal Bone Distance

**Figure 15:**
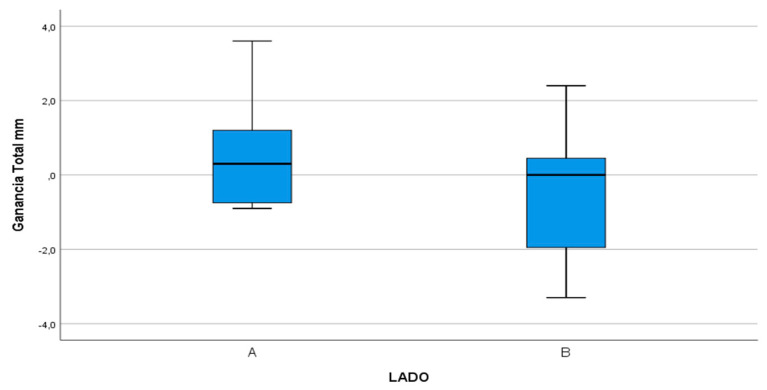
Box-plot representing bone gain in millimeters, measured from the IAN to the bone crest in test group (A) compared with the control group (B).

The distance from the IAN to the bone crest was measured six months after surgery, obtaining 0.54 mm in the test group compared with −0.68 mm in the control group with statistically significant difference (*p* = 0.038). Therefore, the study group showed crestal bone height gain compared to crestal bone loss in the control group (Figure 15).

### 3.5. Pain, Inflammation and Mouth Opening Capacity

The mean surgical time was longer in the test group (26.2 min) compared with the control group (12.38 min). Nevertheless, no significant differences were found between the groups for the variables pain, inflammation, and mouth opening capacity.

**Figure 16:**
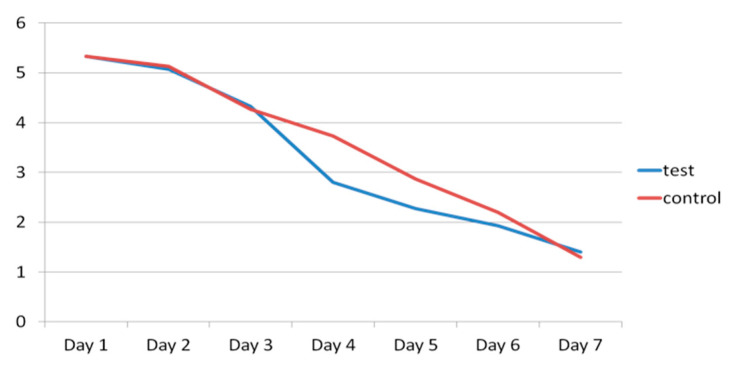
Evolution of pain in test group (blue) and control group (red) for seven days after surgery.

Pain, measured with a VAS, was similar in the test and control groups, with a mean of 5.33 in both groups at the time of surgery, falling to 4.33 vs. 4.27 on the third day after surgery, and 1.40 vs. 1.33 on the seventh day (Figure 16).

**Figure 17:**
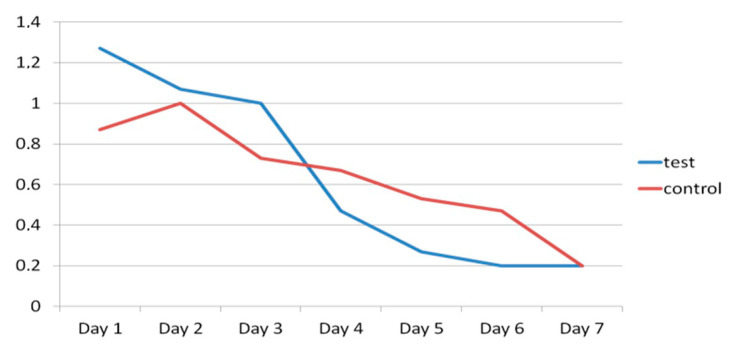
Evolution of rescue analgesics taken in test group (blue) and control group (red) during the seven days after surgery.

As for the numbers of rescue analgesics taken, no significant differences were found between the test and control groups, with a mean number of 1.27 vs. 0.87 on the day of surgery, 1 vs. 0.73 on the third day, and 0.20 in both groups on the seventh day after surgery (Figure 17).

Lastly, inflammation and mouth opening capacity were similar in both groups, with no significant difference between test and control groups. Almost-complete mouth opening capacity was achieved within one week of surgery, and complete capacity was restored within one month.

### 3.6. Intra- and Post-Operative Complications 

Regarding intra-operative complications, one vestibular mucosal tear occurred during mucoperiosteal detachment in the control group.

As for post-operative complications, the same patient suffered a delayed abscess one month later, which was treated with antibiotics (Amoxicilin 750 mg three times a day for a week). A second patient in the control group presented a hematoma in the lower genian region during the post-operative period. A third patient suffered wound dehiscence two weeks after extraction, treated by applying 0.2% chlorhexidine gel (PerioKin^®^, Laboratorios Kin, Barcelona, Spain), which closed the wound.

No sensitive lesions of either the IAN or lingual nerve were produced.

None of these complications presented an obstacle to the patients completing the trial.

## 4. Discussion

This clinical trial found a notable reduction in mean PD in the test group compared with the control group, at both three and six months after surgery, with statistically significant difference between the groups.

Authors such as Leung et al. [3] affirm that the presence of mesioangled LTMs produces a defect on the distal face of the second molar that weakens bone support in the area, forming a periodontal pocket that favors bacterial colonization. At the same time, other authors agree that the surgical extraction of an impacted LTM can also produce a periodontal defect on the distal face of the second molar, and that this defect rarely returns to normality.

In this way, there is some controversy regarding bone gain or loss on the distal side of the lower second molar after impacted LTM extraction, although Kugelberg et al. [24], in a study of LTM extractions, found that 43.3% of cases presented PDs greater than 7 mm, and 32.1% PDs greater than 4 mm two years after extraction surgery. In the present trial, most of the control group sites exhibited pathological PDs (>4 mm) on the distal aspect of the lower second molar, corroborating the data cited above.

To treat these periodontal defects located on the distal aspects of second molars, a root planing technique on the second molar’s distal face was described using curettes or ultrasound to remove the layer of acellular cementum. This solution is based on the fact that LTM extraction leaves the distal root of the second molar exposed, which becomes contaminated by bacteria and toxins, and must be removed meticulously to allow adequate periodontal healing and bone regeneration [25]. Nevertheless, several authors [10] have shown that this technique is insufficient to return periodontal parameters to normal. Although the technique is insufficient by itself, it can be recommended as a coadjuvant to regenerative therapy, as described by most authors [2,10,26] although there are other studies that do not report its use [1,27] and systematic reviews which have not found any benefits derived from the technique following LTM extraction [28]. In the present trial, mechanical debridement was performed in both groups, with pathological PD observed in the control group at the end of the six-month follow-up. Nevertheless, as this is an easy, quick and economical treatment, it may be considered recommendable as a coadjuvant that may bring some benefit.

Other authors make the case for regeneration techniques by means of bone substitutes, guided tissue regeneration, or even procedures applied to soft tissues. It has been demonstrated that guided tissue regeneration with a bone substitute and resorbable or non-resorbable membranes is an effective method for resolving these defects in comparison with conventional LTM extraction without the addition of a graft material [2,5,6,7,10,26].

Following this line of research, systematic reviews by Lee et al. [29] and Camps-Font et al. [30] found that most authors agreed that guided tissue regeneration was more effective when graft materials were used in isolation in terms of the periodontal parameters at the distal aspect of the second molar. In addition, it was noted that there is not yet sufficient scientific evidence to determine which is the best biomaterial to use in this type of defect, and that more clinical trials are needed to answer this question.

Dentin and human bone present many similarities in relation to their inorganic, organic and water content, showing osteoconductive (the hydroxyapatite in dentin takes the form of calcium phosphate with low crystalline content, making it more easily degraded by osteoclasts) [12,31] and osteoinductive properties. Although it does not possess the osteogenic capacity of autogenous bone and its quantity is also limited, the use of autogenous dentin as a graft material exhibits positive behavior and avoids the morbidity and complications associated with harvesting autogenous bone [12].

To date, no other trial has been published that has used autogenous dentin as a graft material to resolve the periodontal defects on the distal aspect of the second molar derived from LTM extraction with post-operative clinical and CBCT monitoring. The present results displayed a significant reduction in probing depth on the test side with dentin graft compared with the control side without grafting during the first three months after surgery, a finding that agrees with a trial conducted by Inocencio-Faria et al. [32] in which the almost-complete healing of the defects was produced during the first three months after surgery.

Regarding the preparation of autogenous dentin for grafting, the present trial used the LTM without removing the enamel, following other authors such as Kabir et al. [20] or Minetti et al. [33] who conducted a clinical trial of alveolar preservation using dentin and enamel. Others such as Valdec et al. [17] removed the enamel, pulp and cementum, while Pohl et al. [34] removed the enamel from the crown and Del Canto et al. [18] and Schwarz et al. [35] used only the roots, having removed the radicular cementum.

In the present trial, a fibrin sponge (Gelatamp^®^) was placed over the autogenous dentin graft to prevent soft tissue invagination, following the method described by Ge et al. [2] who compared the use of autologous bone compared with spontaneous healing after extracting impacted LTMs.

Some authors have reported a relation between surgical time and post-operative pain and inflammation [36]. Although in the present trial, the control side required a mean surgical time of 12.38 min, compared with 26.2 min on the test side, no significant differences in post-operative pain, inflammation and mouth opening capacity were found between the groups at the two-day and seven-day follow-up evaluations.

Kabir et al. [20] employed autogenous dentin for the regeneration of post-extraction alveoli, obtaining a similar radiological aspect to the surrounding bone within 12 months of surgery, evaluated in periapical radiographs and micro-CT scans, as well as higher Hounsfield units in the regenerated area than in native bone. The present trial also observed a similar radiological appearance in native and newly formed bone at six months, and higher Hounsfield units in the test group than the control group. Coinciding with the radiological findings reported by Kabir et al., Schwarz et al. [35] conducted a prospective study of horizontal regeneration techniques using autogenous bone blocks in 15 patients and dentin blocks made from LTMs in another 15 patients. They obtained similar widths and bone densities in both groups, and implants were placed at the regenerated sites 26 weeks after surgery.

Del Canto-Diáz et al. [18] published a split-mouth pilot study, in which alveolar preservation was performed with autologous dentin on the test side, and spontaneous healing on the control side, placing implants 16 weeks later. The distance from the base of the alveolus to the crest of the lingual cortex was calculated, obtaining greater bone loss in the control group than the test group (1.77 vs. 0.42 mm). The present trial measured the distance from the IAN to the crestal area of the vestibular cortex, obtaining favorable results on the test side in comparison with the control side (0.54 vs. −0.68 mm). Del Canto-Diáz et al. also evaluated bone density in coronal, medial and apical areas, obtaining a mean densitometric value of 922.68 HU at 16 weeks in the test group compared with 564.35 HU in the control group, with statistically significant difference, a finding that concurs with the present trial’s results (1538.93 vs. 1122.26 HU) six months after surgery.

The use of autogenous dentin was found to reduce PD and improve insertion levels in periodontal defects after LTM extraction in comparison with non-grafted control sites, showing similar behavior to the other biomaterials investigated to date. Moreover, the dentin grafts obtained higher HU values and bone crest height values in comparison with the control group, while no significant differences between the groups were found for the parameters pain, inflammation, mouth opening capacity or degree of corticalization.

It would be useful to conduct additional studies that compare autologous dentin with other biomaterials to determine which exhibits the most favorable behavior in terms of PD in this type of defect.

## 5. Conclusions

Bone regeneration techniques are recommendable in cases of impacted LTM extraction, because when pathological PDs are produced, manual or ultrasonic debridement is insufficient to restore parameters compatible with health.

Autogenous dentin is a promising graft material for the regeneration of periodontal defects after impacted LTM extraction, as it reduced PD and improved clinical insertion levels in comparison with the conventional surgical approach.

## Figures and Tables

**Table 1:** Pre-operative and intra-operative characteristics in test and control groups.

Group	Test Group (Dentin)	Control Group (Coagulate)
Position	Mesioangular: 6/15 (40%)	Mesioangular: 8/15 (53.4%)
	Horizontal: 3/15 (20%)	Horizontal: 3/15 (20%)
	Vertical: 6/15 (40%)	Vertical: 4/15 (26.6%)
Situation	Partial coverage 5/15 (33.3%)	Partial coverage: 6/15 (40%)
	Total coverage (submucosal): 7/15 (46.7%)	Total coverage (submucosal): 6/15 (40%)
	Total coverage (included): 3/15 (20%)	Total coverage (included): 3/15 (20%)
Surgical difficulty (Parant)	Type 1: 0/15 (0%)	Type 1: 1/15 (6.7%)
	Type 2: 9/15 (60%)	Type 2: 7/15 (46.6%)
	Type 3: 6/15 (40%)	Type 3: 7/15 (46.6%)
	Type 4: 0/15 (0%)	Type 4: 0/15 (0%)
Proximity to the mandibular canal	3/15 (20%)	3/15 (20%)
Surgical time	26.2 min	12.38 min

**Table 2:** Pre-operative and intra-operative characteristics in test and control groups.

Group	Test Group (Dentin)	Control Group (Coagulate)
Patient 1	Vertical	Vertical
	Partial coverage	Partial coverage
	Type 2	Type 1
Patient 2	Mesioangular	Mesioangular
	Total coverage (submucosal)	Total coverage (submucosal)
	Type 2	Type 2
Patient 3	Horizontal	Horizontal
	Partial coverage	Total coverage (submucosal)
	Type 3	Type 3
Patient 4	Mesioangular	Mesioangular
	Total coverage (submucosal)	Partial coverage
	Type 2	Type 2
Patient 5	Mesioangular	Mesioangular
	Total coverage (submucosal)	Partial coverage
	Type 3	Type 3
Patient 6	Horizontal	Horizontal
	Total coverage (included)	Total coverage (included)
	Type 3	Type 3
Patient 7	Vertical	Mesioangular
	Total coverage (included)	Total coverage (included)
	Type 3	Type 3
Patient 8	Mesioangular	Mesioangular
	Total coverage (submucosal)	Total coverage (submucosal)
	Type 3	Type 3
Patient 9	Mesioangular	Mesioangular
	Total coverage (submucosal)	Total coverage (submucosal)
	Type 2	Type 2
Patient 10	Horizontal	Vertical
	Total coverage (included)	Total coverage (included)
	Type 3	Type 3
Patient 11	Vertical	Mesioangular
	Total coverage (submucosal)	Total coverage (submucosal)
	Type 2	Type 2
Patient 12	Vertical	Mesioangular
	Partial coverage	Total coverage (submucosal)
	Type 2	Type 2
Patient 13	Vertical	Horizontal
	Partial coverage	Partial coverage
	Type 2	Type 3
Patient 14	Vertical	Vertical
	Total coverage (submucosal)	Partial coverage
	Type 2	Type 2
Patient 15	Mesioangular	Vertical
	Partial coverage	Partial coverage
	Type 2	Type 2
